# Supplementary material for: The Role of TLR-2 in Lethal COVID-19 Disease Involving Medullary and Resident Lung Megakaryocyte Up-Regulation in the Microthrombosis Mechanism
Source: Cells. 2024 May 17;13(10):854. doi: 10.3390/cells13100854 (PMC11120208; doi:10.3390/cells13100854)
Supplement: Supplementary file 1 [file cells-13-00854-s001.zip › cells-2947345-supplementary.pdf]

Supplementary materials

Table S1. ***Patients.*** A series of 25 patients with someone comorbidities (Chronic nephropathy, Hypertension, cardiac hypertrophy, Obesity, diabetes mellitus, Obstructive chronic broncho-pneumopathy) who died from a lethal form of Sars-CoV2 infection were studied. 13 males and 12 females, age between 45 and 80 years.

	Age < 50	Age >50
**Female COVID-19 cases**	1	11
**Female Control cases**	1	2
**Male Covid-19 cases**	2	11
**Male Control cases**	1	7

Table S2. Summary of clinic-pathological parameters of patients groups according to COVID-19 status.

VARIABLES	COVID-19 STATUS	
	POSITIVE (total n.25)	*NEGATIVE* (total n.11)
**Age, Mean ± SEM**	65,76 ± 2,11	68.5 ± 5,40
**Gender, Male/Female (%)**	13/12 (1,08)	8/3 (2,66)
**Pathological co-morbidities, Yes/Not (%)**	24/1 (96)	11/11 (100)
**Single co-morbidity/morbidity, (%)**	12/25 (48)	11/11 (100)
**Type of single co-morbidities/morbidity, (%)**	Hypertension 18/25 (72)	Lung metastatic carcinoma 2/11(18,18)
	Cardiac Hypertrophy 8/25 (32)	Primary lung neoplasm 6/11 (54,54)
	OCBP, 3/25 (12)	Lung emphysema 2/11 (18,18)
	Chronic nephropathy 2/25 (8)	Lung hamartoma 1/11 (9,09)
	Obesity 5/25 (20)	0/11 (0)
**Multiple co-morbidities/morbidity, (%)**	12/25 (48)	0/11(0)
**Type of multiple co-morbidities/morbidity, (%)**	Hypertension; CH, Cardiac Hypertrophy 6/25 (24)	
	Obesity, and NIDDM 2/25 (8)	
	Obesity, hypertension, and NIDDM 1/25 (4)	
	Hypertension; CH, Cardiac Hypertrophy, and Chronic nephropathy 7/25 (28)	
	Obesity, Hypertension, and Cardiac Hypertrophy 1/25 (4)	

Legend. NIDDM, Type II- *Non-Insulin-Dependent Diabetes Mellitus;* OCBP, Obstructive Chronic Broncho-Pneumopathy; CN, Chronic Nephropathy; O, Obesity; H, Hypertension; CH, Cardiac Hypertrophy.

Table S3. Antibody used and experimental conditions.

ANTIBODY	CLONE	METHOD
TLR2	rabbit policlonal	LSAB-HRP/AP, Ventana Benchmark® XT autostainer

CD61	2f2	LSAB-HRP/AP, Ventana Benchmark® XT autostainer
